# Real-time optotracing of curli and cellulose in live *Salmonella* biofilms using luminescent oligothiophenes

**DOI:** 10.1038/npjbiofilms.2016.24

**Published:** 2016-11-23

**Authors:** Ferdinand X Choong, Marcus Bäck, Sara Fahlén, Leif BG Johansson, Keira Melican, Mikael Rhen, K Peter R Nilsson, Agneta Richter-Dahlfors

**Affiliations:** 1 Swedish Medical Nanoscience Center, Department of Neuroscience, Karolinska Institutet, Stockholm, Sweden; 2 Division of Chemistry, Department of Physics, Chemistry and Biology, Linköping University, Linköping, Sweden; 3 Department of Microbiology, Tumor and Cell biology, Karolinska Institutet, Stockholm, Sweden

## Abstract

Extracellular matrix (ECM) is the protein- and polysaccharide-rich backbone of bacterial biofilms that provides a defensive barrier in clinical, environmental and industrial settings. Understanding the dynamics of biofilm formation in native environments has been hindered by a lack of research tools. Here we report a method for simultaneous, real-time, *in situ* detection and differentiation of the *Salmonella* ECM components curli and cellulose, using non-toxic, luminescent conjugated oligothiophenes (LCOs). These flexible conjugated polymers emit a conformation-dependent fluorescence spectrum, which we use to kinetically define extracellular appearance of curli fibres and cellulose polysaccharides during bacterial growth. The scope of this technique is demonstrated by defining biofilm morphotypes of *Salmonella enterica* serovars Enteritidis and Typhimurium, and their isogenic mutants in liquid culture and on solid media, and by visualising the ECM components in native biofilms. Our reported use of LCOs across a number of platforms, including intracellular cellulose production in eukaryotic cells and in infected tissues, demonstrates the versatility of this optotracing technology, and its ability to redefine biofilm research.

## Introduction

Biofilms are a natural multicellular form of bacterial life, which contribute to resistance against antibiotics, the host immune systems and environmental stresses.^1^ Biofilms enable bacteria to colonise abiotic surfaces (e.g., stainless steel,^2^ glass^3,4^ and plastics^5^) as well as biotic surfaces, such as epithelial cells and other tissue compartments.^6,7^ Embedded in an endogenously produced extracellular matrix (ECM), these mono- or poly-bacterial populations are difficult to eradicate. Although the overall dry mass of the biofilm may be substantial, microbial cells only constitute a small fraction, with the majority attributed to the extracellular polymeric matrix.^8^ The composition of ECM varies, but adhesins, amyloid-forming proteins and extracellular polysaccharides are ubiquitous ECM components.^9^ The amyloid curli fimbriae and bacterially produced cellulose have been identified as important ECM components for *Escherichia coli*, and *Salmonella enterica* serovars Enteritidis (*S.* Enteritidis) and Typhimurium (*S.* Typhimurium).^10,11^

Existing methods for biofilm detection and quantification are largely based on colorimetric assays using Crystal violet (CV), Congo red (CR) and Thioflavin derivatives. The CV assay, based on retention of molecules by hydrostatic interactions, provides only an indirect measure of biofilm,^12^ whereas the CR, Thioflavin and other hydrophobic molecules, which bind to ECM polysaccharides and amyloid proteins, enable direct quantification using fluorometric signals.^13,14^ The chemical nature of current dyes restricts their use to end-point measurements, with toxicity hindering their application in real-time studies of biofilm formation *in vitro* and *in vivo.*

In the research field of Alzheimer’s disease, luminescent conjugated oligothiophenes (LCOs) have been used to detect amyloid protein aggregates both *in vitro* and *in vivo.*^15–17^ Surpassing the conventional amyloid ligands CR and Thioflavin, LCOs identify a broader subset of disease-associated protein aggregates and enable spectroscopic assignment of heterogeneous populations of deposits.^18–21^ The flexible conjugated thiophene backbone distorts in response to non-covalent electrostatic interactions with target molecules. This generates a conformation based, target specific, spectral signature, which in contrast to conventional fluorophores, exhibits an ON/OFF fluorescent signature.^15–17^ Interactions with amyloid proteins characteristically lead to the flattening of the molecular backbone and a more effective conjugation, causing a red-shift in the fluorescence excitation as well as increased fluorescence emission intensity.^15–17^ The excitation spectrum in particular is a direct reflection of the LCO backbone geometry.

Since amyloid proteins and fibrous polysaccharides are major ECM constituents, we hypothesised a novel use of LCOs as non-bactericidal, conformational sensitive fluorescent probes for real-time detection and differentiation of these essential components of *Salmonella* biofilms, a method we define as optotracing. To our knowledge, no conventional dyes, or other available techniques are able to monitor dynamic biofilm formation and concurrently differentiate between curli fibres and cellulose under live conditions. Visualising the dynamics of biofilm formation under live conditions at a resolution where individual biofilm components are detected is thus hampered. To address this need, we employed two prototype, non-toxic LCO molecules to dynamically detect and differentiate between curli fibres and cellulose polysaccharides in *S.* Enteritidis and *S.* Typhimurium forming biofilm on abiotic surfaces, agar plates, in liquid cultures, intracellularly in eukaryotic cells, and in mouse liver.

## Results

### Fluorescent differentiation of ECM components using luminescent oligothiophenes

**Figure 1:**
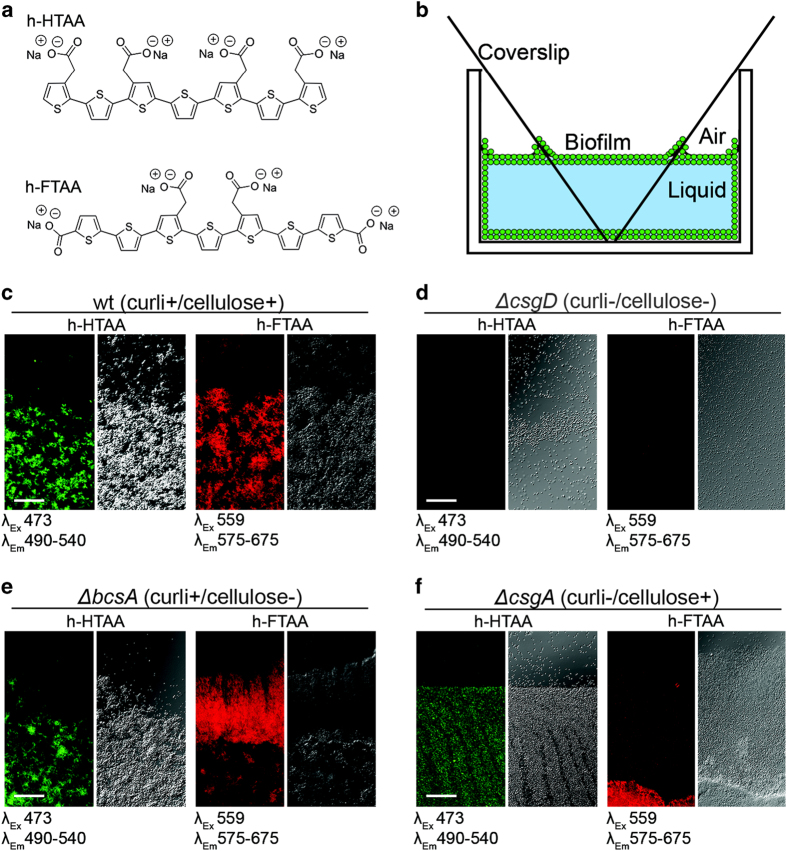
LCO staining patterns distinguish *Salmonella* biofilms. (**a**) Structure of h-HTAA and h-FTAA. (**b**) Schematic of the incline glass coverslip setup enabling microscopic analysis of biofilm at air–liquid interface after removal of coverslips. (**c**–**f**) Fluorescence confocal microscopy using indicated excitation and emission wavelengths (left) and transmission confocal microscopy (right) of h-HTAA- and h-FTAA-stained biofilms from strains 3934 (**c**) wt, (**d**) Δ*csgD*, (**e**) Δ*bcsA* and (**f)** Δ*csgA* with indicated curli and cellulose phenotypes. Single optical sections are shown. Scale bar=50 μm.

Two LCOs, h-HTAA and h-FTAA (Figure 1a), selected from our library of synthesised LCO molecules for their amyloid sensitivity, were screened for their suitability as optotracers of biofilm ECM components on an isogenic collection of *S.* Enteritidis based on the wild-type (wt) strain 3934 (Supplementary Table 1). To facilitate analysis of surface-bound biofilm formed at the air-liquid interface, bacteria were grown in wells with inclined square glass coverslips (Figure 1b). After gentle removal of the coverslips, LCOs were first applied directly onto the surfaces, which then were prepared for microscopic analysis. Fluorescence microscopy of the biofilms demonstrated distinct labelling, suggesting that h-HTAA (green) and h-FTAA (red) fluorescence signals can complement phase contrast when visualising biofilm morphology (Figure 1c and Supplementary Figure 1). In contrast, no fluorescent signals were identified from a Δ*csgD* mutant strain unable to produce curli and cellulose (Figure 1d). Individual contribution of the two ECM elements to the positive LCO-biofilm staining was analysed using Δ*bcsA* (curli^+^ cellulose^−^) and Δ*csgA* (curli^−^ cellulose^+^) mutant strains. Phase contrast microscopy of the cellulose-deficient mutant (strain Δ*bcsA*) showed similar morphology to the wt, and distinct fluorescence signals from both LCOs (Figure 1e). A thin and brittle bacterial layer, typically formed in the absence of curli,^22^ was observed in the Δ*csgA* mutant strain and LCO staining revealed distinct fluorescence that was more pronounced in areas with higher cell density (Figure 1f).

**Figure 2:**
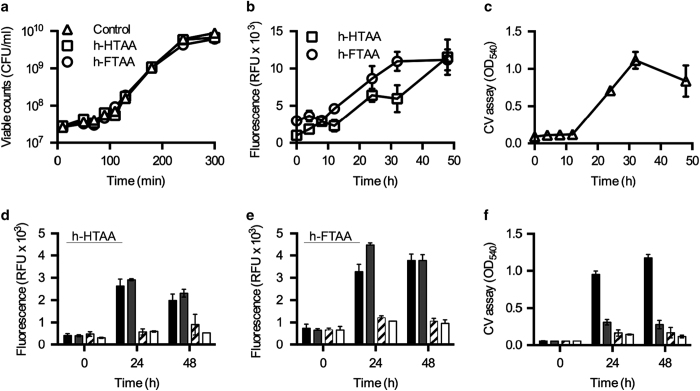
LCO-based fluorometric biofilm quantification in a small-volume 96-well assay. (**a**) Growth curve, shown as viable counts, of strain 3934 wt cultured in the absence (control) and presence of h-HTAA and h-FTAA. (**b** and **c**) End point quantification of biofilm formed by 3934 wt at indicated times based on (**b**) fluorescence from cultures grown in the presence of h-HTAA and h-FTAA and (**c**) the crystal violet assay. (**d**–**f**) Quantification of biofilm formed at 24 and 48 h by 3934 wt (■), Δ*bcsA* (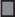), Δ*csgA* (▨) and Δ*csgD* (□) based on fluorescence from (**d**) h-HTAA, (**e**) h-FTAA, and based on the (**f**) crystal violet assay. Data represent *n*: 1 of 3 with standard deviations shown. CFU, colony forming units; CV, crystal violet; RFU, relative fluorescence units.

Growth curves of the wt strain in the absence and presence of LCOs revealed a common generation time of 23±1 min, indicating that neither h-HTAA nor h-FTAA exert bacteriostatic or bactericidal effects (Figure 2a). This corroborated previous demonstrations of a non-toxic nature of the oligothiophene family on eukaryotic cells *in vitro* and in intravital mouse models.^23,24^ We therefore tested LCOs in live cultures, to monitor the extracellular appearance of curli fibres and cellulose during growth. A custom-designed, small-volume 96-well assay (Supplementary Figure 2) enabled analysis of pellicle and surface-attached biofilm at selected time points using spectrophotometric recordings in a standard plate reader. Fluorescence analysis of wt cultures in the presence of h-HTAA or h-FTAA (Figure 2b) showed a comparable increase of biofilm growth to parallel CV assays (Figure 2c). We next addressed if LCOs can distinguish between different biofilm phenotypes by analysing wt and mutant biofilm formation in the small-volume 96-well assay. Fluorometric read-outs (excitation wavelength (*λ*_Ex_) of 405 nm and emission wavelength (*λ*_Em_) of 556 nm) taken at set time points from a continuous culture grown in the presence of h-HTAA or h-FTAA, revealed that the wt and Δ*bcsA* (curli^+^ cellulose^−^) strains produced high quantities of biofilm (Figure 2d,e). In contrast, Δ*csgA* (curli^−^ cellulose^+^) and Δ*csgD* (curli^−^ cellulose^−^) mutants produced only low amounts. These results supported the microscopy-based demonstration of the Δ*bcsA* mutant as a substantial biofilm producer (Figure 1e). This result differed however from traditional CV assay results, which showed poor biofilm formation by all three mutants (Figure 2f). LCO-based detection appears to differentiate between biofilm phenotypes, with substantially improved sensitivity in biofilms with curli as the major ECM component.

### Non-disruptive analysis of biofilm formation

**Figure 3:**
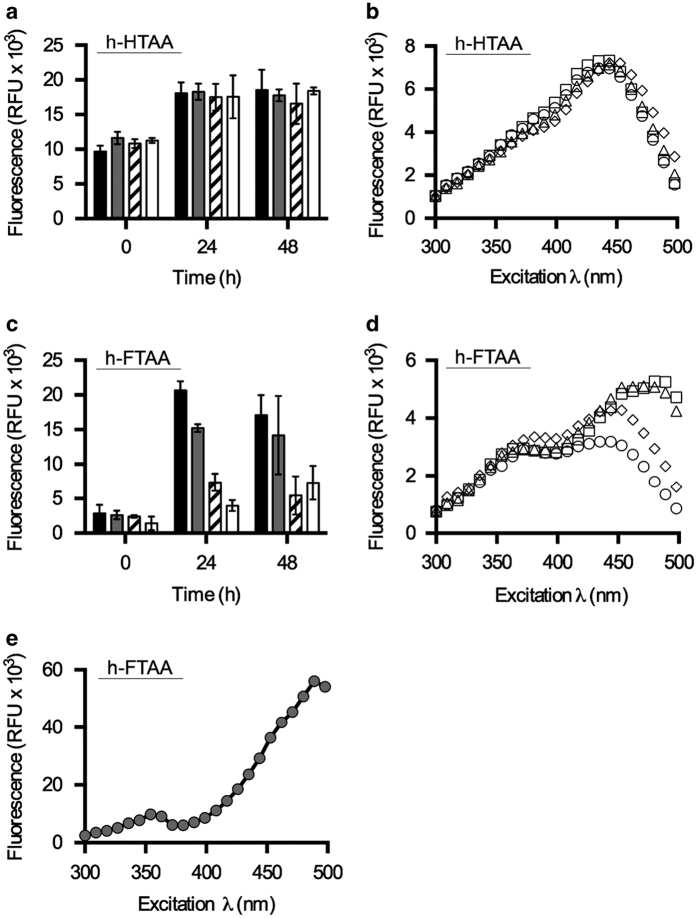
Individual and simultaneous LCO-based quantification of curli and cellulose in longitudinal biofilm cultures. (**a**) Fluorescence of h-HTAA at 24 and 48 h in non-disrupted liquid cultures of 3934 wt (■), Δ*bcsA* (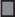), Δ*csgA* (▨) and Δ*csgD* (□). (**b**) Spectra of h-HTAA in cultures at 24 h of 3934 wt (△) Δ*bcsA* (◊), Δ*csgA *(□) and Δ*csgD* (○), with emission read at 545 nm. (**c**) Fluorescence of h-FTAA at 24 and 48 h in non-disrupted liquid cultures of 3934 wt (■), Δ*bcsA* (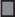), Δ*csgA* (▨) and Δ*csgD* (□). (**d**) Spectra of h-FTAA at 24 h of 3934 wt (△)Δ*bcsA* (◊), Δ*csgA* (□) and Δ*csgD* (○), with emission read at 545 nm. (**e**) Spectra of h-FTAA mixed with cellulose (5 mg/ml) with emission read at 545 nm. Data represent *n*: 1 of 3 with standard deviations shown in **a** and **c**. RFU, relative fluorescence units.

When studying the kinetics of biofilm formation, longitudinal experiments should ideally be performed with minimum disturbance, such as washing procedures. To test whether LCOs can be used for non-disruptive studies, strains were cultured in h-HTAA-supplemented medium in 96-well plates, and the fluorescence of each well was directly recorded at 0, 24 and 48 h. A 4–6-fold fluorescence increase was observed across all strains (Figure 3a) compared with equivalently washed, end-point experiments (Figure 2d). The signals, however, no longer distinguished between the biofilm phenotypes, suggesting that the complex composition of the LB culture contributes to a high background, emitting in the same wavelength window as h-HTAA. To test this hypothesis, excitation spectra from each 24 h culture were collected. Identical spectra, sharing one characteristic peak, were observed for all strains irrespective of the amounts and phenotypes of the biofilms (Figure 3b). This suggested that h-HTAA binds a biofilm component different from curli and cellulose, which is ubiquitously present in all cultures. While limited in use for the purpose of the present study, h-HTAA still represents an interesting alternative to current biofilm dyes as a general non-bacteriocidal, fluorescence alternative for endpoint studies.

The h-FTAA was, however, able to differentiate biofilm phenotypes in the longitudinal assays (Figure 3c). With h-FTAA, the cellulose-deficient mutant Δ*bcsA* showed significant amounts of biofilm, with a 4–5 fold increase in signal intensity at 24 h, a similar pattern to the washed endpoint experiments (Figure 2e). No further increase was observed, indicating that the bulk of this curli-based biofilm had formed in the first 24 h. Despite high background, spectral analysis showed increased fluorescence intensity from curli-producing wt and Δ*bcsA* compared with the curli-deficient strains at *λ*_Ex_ 405 nm, which may represent a signal unique to h-FTAA bound to curli fibres (Figure 3d). A unique excitation peak also appeared at ~480 nm in wt and Δ*csgA* (curli^−^ cellulose^+^) strains suggesting the simultaneous detection of a second target.

Based on the genetic make-up of our *Salmonella* strains, cellulose, whose biosynthesis is a shared trait in the wt and Δ*csgA* (curli^−^ cellulose^+^) strains appears to be a strong candidate to serve as a second binding target for h-FTAA. To test this hypothesis, the excitation spectra of h-FTAA combined with different concentrations of pure cellulose was obtained. The spectra showed an evident peak with *λ*_max_ at ~480 nm when emission was recorded at 545 nm (Figure 3e (5 mg/ml) and Supplementary Figure 3 (0.04–2.5 mg/ml). This peak is comparable to that generated by the cellulose-producing *Salmonella* strains (Figure 3d), and demonstrates the exclusive spectral signature of h-FTAA binding to cellulose. This finding extends the applicability of LCOs from conformation-sensitive spectral probes detecting amyloid protein aggregates^15–17^ to also include polysaccharides.

### Spectral morphotyping of biofilm

**Figure 4:**
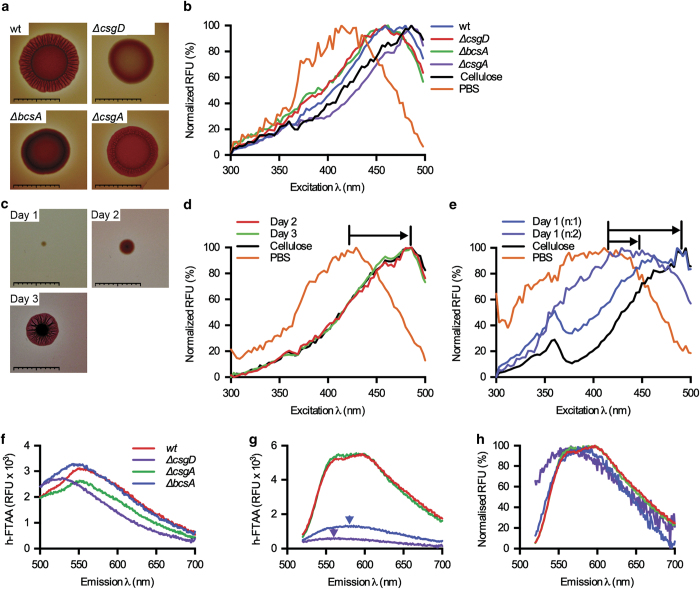
LCO-based morphotyping of *Salmonella* biofilms from agar plates. (**a**) Morphotypes of strain 3934 wt, Δ*csgD*, Δ*bcsA* and Δ*csgA* based on the drop assay on Congo red plates. (**b**) Normalised spectra of h-FTAA mixed with re-suspended biofilm colonies harvested from indicated strains grown for 48 h on LB agar w/o salt, with emission read at 545 nm. h-FTAA mixed with cellulose and PBS were assayed in parallel for reference. (**c**) Morphotype of a 3934 wt biofilm colony originating from an individual bacterium on Congo red plates monitored for three consecutive days. (**d** and **e**) Spectra of h-FTAA mixed with harvested 3934 wt biofilm colonies at (**d**) days 2 and 3, and (**e**) day 1, including cellulose and PBS for reference. Arrows indicate the shift in *λ*_max_ for h-FTAA in the presence of various amounts of cellulose. *n*: 1 of 5 in **b** and **d**, *n*: 2 of 5 in **e**. Scale bars=1 cm. (**f**) Emission spectra of h-FTAA-supplemented cultures of strain 3934 wt, Δ*bcsA*, Δ*csgA* and Δ*csgD* after 24 h incubation, using excitation at 405 nm for curli detection. (**g**) Same experimental setup as in **f** using excitation at 500 nm for cellulose detection. Arrows indicate *λ*_max_ of emission in Δ*csgA* and Δ*csgD* mutant strains. (**h**) Normalised fluorescence spectra for cellulose detection from **g**. Data represent *n*: 1 of 3. RFU, relative fluorescence units.

More than 90% of *S.* Typhimurium and *S.* Enteritidis strains produce a characteristic red, dry and rough colony morphology on solid media, the so-called rdar morphotype.^25^ This three-dimensional architecture is formed by highly ordered spatial arrangement of cellulose filaments and curli fibre networks.^26^ We analysed whether LCOs can be used for definitive spectral morphotyping of bacterial colonies. Having ascertained that the strains showed the expected rdar morphotypes on CR plates^27^ (Figure 4a), we added h-FTAA to re-suspensions of 3-day-old colonies grown on LB agar plates without salt, and performed spectral analysis. Comparison of differently sized colonies at different developmental stages was enabled by normalising each spectrum such that each data point is represented as a percentage of the largest emitted fluorescence of the excitation spectra. A distinct red-shifted cellulose-specific peak was observed at 480 nm in wt and Δ*csgA* (curli^−^ cellulose^+^) colonies, overlapping the signal from h-FTAA binding pure cellulose (Figure 4b). The peak was absent in Δ*csgD* and Δ*bcsA* colonies, both genetically incapable of cellulose production.

Rdar morphotyping on CR plates requires that a bacterial colony, often initiated from a 25 μl drop of ~10^5^ c.f.u., reach a sufficient size for visual inspection, which usually takes 3 days (Figure 4c). To enable non-biased profiling during biofilm development on LB plates without salt, we analysed daily harvests of individual wt colonies, originating from a single bacterium, for the presence of cellulose. Spectra of colony re-suspensions stained with h-FTAA consistently showed a cellulose peak at 480 nm on day 2, and persisting on day 3 (Figure 4d). The presence of this peak was less consistent on day 1 (Figure 4e). The variability at this early stage reflects a changing ratio of unbound and bound h-FTAA as the amount of cellulose in the colony increases during biofilm maturation. In some day 1 colonies, cellulose was produced which bound all h-FTAA molecules, thus generating the 480 nm peak (Figure 4e, long arrow). In colonies with less cellulose, the higher proportion of unbound h-FTAA was observed as an intermediate degree of red-shifted *λ*_max_ (Figure 4e, short arrow). The transition of *λ*_max_ reflects cellulose production during the transition between different bacterial growth stages of colonies growing on solid medium. Such transient events are invisible to eye inspection (Figure 4c).

### Defining optical settings for simultaneous, dual detection of cellulose and curli

The binding of h-FTAA to amyloid curli protein and cellulose suggests its possible application as an optotracer for simultaneous, dual detection of ECM components. We applied the small-volume 96-well assay of defined bacterial cultures to test whether we could identify discrete spectral signatures for each target. To identify the optimum emission wavelength (*λ*_Em_) of h-FTAA bound to curli, we collected the emission spectra with excitation at 405 nm. The three biofilm-forming strains produced red-shifted emission peaks at *λ*_max_ 550–560 nm, compared with a *λ*_max_ 525 nm in the non-biofilm forming Δ*csgD* mutant (Figure 4f). Increased fluorescence amplitude was only observed in curli-containing biofilms (wt and Δ*bcsA*), corroborating the finding in Figure 3c. This suggests *λ*_Em_ 550–560 nm as an optimum range for curli detection. Cellulose does not appear to contribute to the background signal, since the red-shift appearing in the cellulose-expressing Δ*csgA* curli mutant was not associated with a fluorescence intensity increase.

To define the optimal emission wavelength for cellulose detection, excitation at 500 nm was used, as this wavelength was observed to maximise the signal-to-background ratio (Figure 3d). Two prominent emission peaks at ~560 nm and ~597 nm were seen for h-FTAA binding to the cellulose-containing biofilm from wt and Δ*csgA* (Figure 4g). Strains lacking this ECM component (Δ*bcsA* and Δ*csgD*) showed h-FTAA binding to curli or an unknown component, inferred from the weak *λ*_max_ signals at 566 and 586 nm that were only noticeable following data normalisation (Figure 4h). Detection above 597 nm in cellulose-producing strains resulted in less background than detection at 560 nm. Ensuring minimal contribution of background, optimal spectral parameters for h-FTAA based cellulose detection were identified as *λ*_Ex_ 500 nm and *λ*_Em_ 600 nm. Combining this with spectral parameters optimal for curli (*λ*_Ex_ 405 nm, *λ*_Em_ 556 nm) thus enables dual detection of the ECM components.

### Real-time detection of curli and cellulose in liquid bacterial cultures

**Figure 5:**
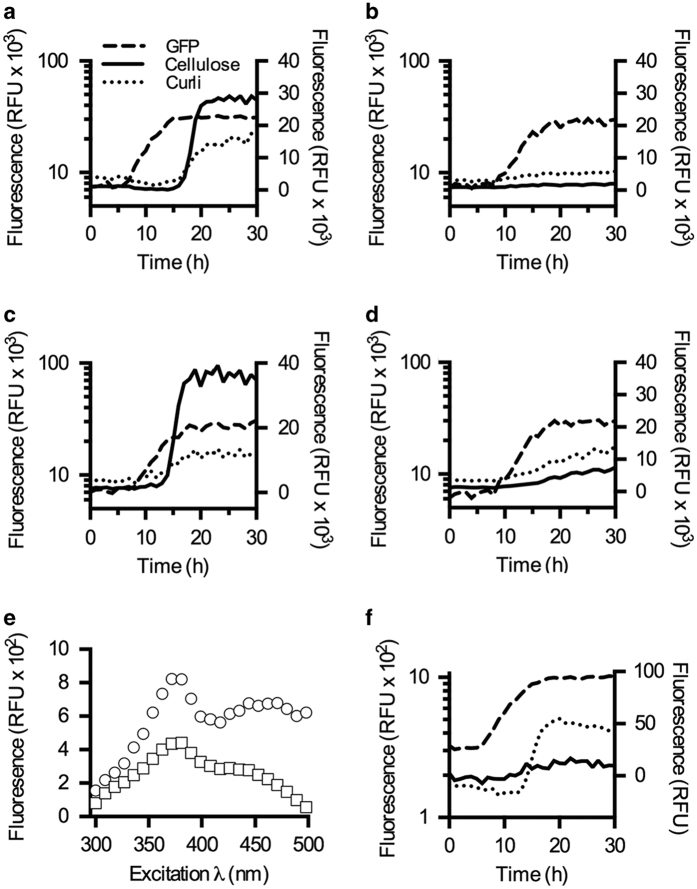
h-FTAA enables simultaneous, real-time detection of curli and cellulose in liquid *Salmonella* cultures. (**a**–**d**) Combined real-time recording of bacterial growth (left *y*-axis) measured by intensity of GFP (*λ*_Ex_ 445 nm, *λ*_Em_ 510 nm), and h-FTAA staining extracellular curli (*λ*_Ex_ 405 nm, *λ*_Em_ 556 nm) and cellulose (*λ*_Ex_ 500 nm, *λ*_Em_ 600 nm; right *y*-axis) in liquid cultures of strains 3934 (**a**) wt, (**b**) Δ*csgD*, (**c**) Δ*csgA* and (**d**) Δ*bcsA* harbouring plasmid p2777. (**e**) Autofluorescence from LB medium only (□), as well as the combined background fluorescence from biofilm-forming, GFP-expressing bacteria in LB medium (○). (**f**) Combined real-time recording of bacterial growth, monitored by GFP expression (dashed line, left *y*-axis) from strain 3934 wt p2777, and appearance of cellulose detected by h-FTAA (right *y*-axis) in the absence (dotted line) and presence (solid line) of the cellulose-digesting enzyme cellulase.

In contrast to traditional fluorophores, fluorescence from the LCOs is modulated by the molecular geometry. As the geometry changes in the bound versus unbound state, fluorescence intensity increases at a given wavelength, showing an ON-like switch as the corresponding binding target appears. We evaluated LCOs as dynamic optotracers of curli and cellulose during biofilm formation, using spectrophotometric recordings of h-FTAA added to bacterial cultures in the small-volume 96-well format. Bacterial GFP expression (plasmid p2777—Supplementary Table 1) enabled simultaneous recording of bacterial growth based on GFP expression (*λ*_Ex_ 445 nm, *λ*_Em_ 510), and h-FTAA detection of curli (*λ*_Ex_ 405 nm, *λ*_Em_ 556 nm) and cellulose (*λ*_Ex_ 500 nm, λ_Em_ 600 nm). The appearance of the curli and cellulose spectral signatures coincided with the shift from late logarithmic to early stationary phase at circa 15 h in the wt culture (Figure 5a). Significant signal increase demonstrated pronounced secretion and assembly of both ECM components during early stationary phase. After ~5 h, production ceased and the signals remained at a consistent level throughout late stationary phase. No signal was detected in the curli- and cellulose-deficient strain Δ*csgD*-p2777 (Figure 5b).

In the curli-deficient Δ*csgA*-p2777 strain, cellulose was expressed 4 h earlier than the wt culture, during the exponential growth phase (Figure 5c). Multiple comparison analysis (ANOVA) revealed that this difference was statistically significant (Supplementary Figure 4 and Supplementary Table 2). The sharp increase leveled off, and remained constant from early stationary phase onwards. In the curli detection window, a small non-specific signal appeared, which we could ascribe to the broad emission range of h-FTAA bound to cellulose (Figure 4g).

The cellulose-deficient strain Δ*bcsA*-p2777, showed a slight signal in the cellulose detection window (Figure 5d), which was identified as GFP ‘bleed-through’ by an excitation scan of a planktonic GFP-producing culture (Figure 5e). Curli production increased only gradually throughout the time course of an experiment, a trend differing from the conventional sigmoidal dynamics of ECM formation (Figure 5a). These data suggest that the absence of cellulose de-regulates the production of curli fibres. The kinetics of immediate LCO fluorescence upon amyloid assembly^28^ implies that the gradual signal increase directly reflects the appearance of curli in the biofilm. The topic of cellulose- and curli co-regulation in biofilm needs further study, studies in which LCOs can play a major role.

To verify the specificity of h-FTAA as a tool for real-time *in situ* measurements of assembled biofilm components, we grew the wt-p2777 strain in the presence a cellulose-degrading enzyme. Whereas increased GFP fluorescence indicated bacterial growth was not affected by the addition of cellulase, the h-FTAA signal was suppressed due to the effective elimination of the cellulose polysaccharides (Figure 5f). Taken together, our data show h-FTAA to be able to specifically detect the expression of cellulose and curli simultaneously in a continuously growing culture, opening up many exciting possibilities for further kinetic studies.

### Fluorescence imaging of biofilm ECM

**Figure 6:**
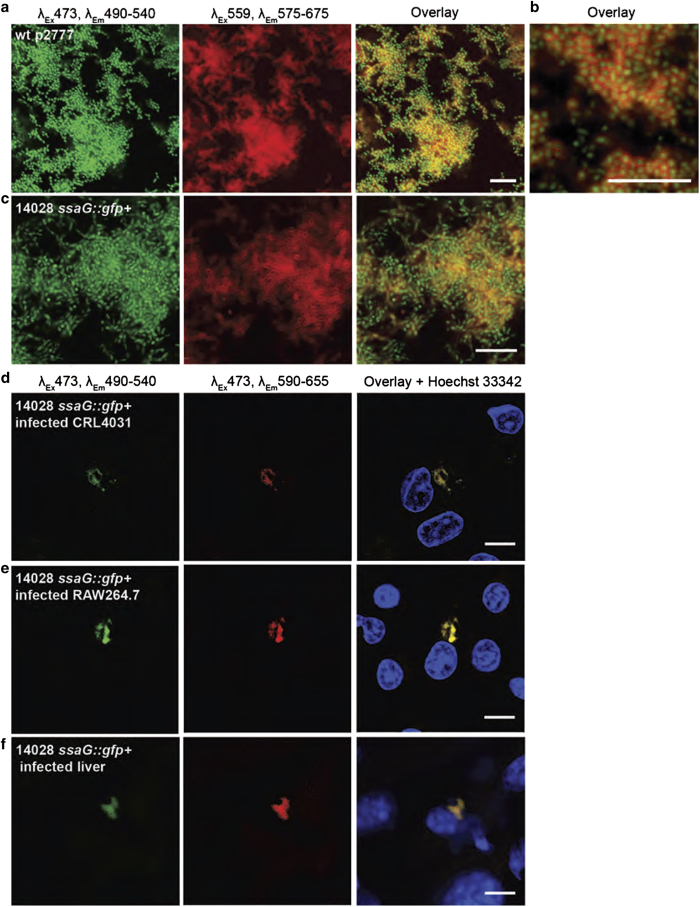
Visualisation of ECM components in biofilms formed by *S.* Enteritidis and *S.* Typhimurium in the presence of h-FTAA. (**a**–**c**) Fluorescence confocal microscopy of unfixed biofilm formed by *S.* Enteritidis strain 3934 wt p2777 at (**a**) lower and **(b**) higher magnification, as well as (**c**) *S.* Typhimurium strain 14028 *ssaG:gfp^+^* during growth on inclined coverslips in medium supplemented with h-FTAA. Bacteria (green) and ECM (red) are detected at indicated wavelengths, representing the microscopes’ pre-defined detection settings for GFP and Cy3. (**d**–**f**) Fluorescence confocal microscopy images of 14028 *ssaG:gfp^+^* (green) infected (**d**) CRL-4031 epithelial cells, and (**e**) RAW264.7 macrophage cell and in (**f**) sections of mouse livers stained with h-FTAA (red). Staining with Hoechst 33324 shows nuclei (blue) of each cell type. Single optical sections are shown. Scale bar=10 μm.

Fluorescence-based microscopy techniques are fundamental tools for the better understanding of biological events. As a lab with a strong interest in microscopy, we analysed the use of LCOs for *in situ* staining of ECM in growing biofilms. Using the inclined coverslip set-up, cultures of wt-p2777 in medium supplemented with h-FTAA-enabled incorporation of the optotracer molecules into biofilm formed at the air–liquid interface of the coverslips. Without any additional treatment, confocal microscopy was performed directly on coverslips removed at 24 h. Large communities of distinct rod-shaped GFP-expressing bacteria were observed, surrounded by dense mesh-like structures visualised by fluorescence from h-FTAA bound to the ECM (Figure 6a and Supplementary Figure 5a,b). The fluorescence of h-FTAA was detectable using standard microscopy settings for Cy3 (*λ*_Ex_ 559 nm, *λ*_Em_ 575–675 nm). Higher magnification revealed the spatial relationship more clearly, showing red ECM filling the extracellular spaces between green bacterial cells (Figure 6b). No signs of intracellular h-FTAA binding were observed.

### Intracellular cellulose detected by h-FTAA in *S.* Typhimurium infection

Biofilm is considered an important factor in the pathogenesis of *Salmonella* infections. Shifting to the common model serovar *S.* Typhimurium, we first confirmed that its ECM components are also detectable by h-FTAA. Fluorescent spectral profiling of biofilm formed by the *S.* Typhimurium strain 14028 *ssaG::gfp*^*+*^ (Supplementary Table 1) showed the same distinctive emission spectra as *S.* Enteritidis, with a signature cellulose peak at 480 nm (Supplementary Figure 6a). h-FTAA also proved useful for kinetic, real-time recordings of curli and cellulose production in growing cultures (Supplementary Figure 6b), and for microscopy-based visualisation of the mesh-like ECM in *S.* Typhimurium strain 14028 *ssaG::gfp*^*+*^ (Figure 6c). Due to structural similarities, the cellulose polysaccharide can thus be successfully identified by h-FTAA across different *S.* Enterica serovars.

*S.* Typhimurium is a facultative intracellular pathogen that proliferates in phagocytic and non-phagocytic cells.^29^ We analysed whether h-FTAA could identify cellulose produced during the intracellular life cycle of 14028 *ssaG::gfp*^*+*^. Infected epithelial cells and macrophages were incubated in h-FTAA, then analysed under confocal microscopy. Using optical settings optimised for cellulose detection (*λ*_Ex_ 473 nm, *λ*_Em_ 550–655 nm), red fluorescence was observed that co-localised to GFP-expressing bacteria (Figure 6d,e, Supplementary Movies 1 and 2). These data were extended to tissue infection, where cellulose stained by h-FTAA was observed in association with bacteria inside the liver of infected mice (Figure 6f). Collectively, our data demonstrate biofilm formation as an integrated feature of *Salmonella*’s intracellular lifestyle, and corroborates recent findings demonstrating cellulose production by *Salmonella* inside macrophages.^30^

## Discussion

The essential role of ECM in establishing sessile and planktonic biofilms is well recognised.^31,32^ Expression of ECM is dynamically and environmentally regulated,^33,34^ highlighting the importance of monitoring production of these components in real-time in the endogenous environment. The current lack of methods for direct labelling and discrimination of ECM components under live conditions has severely hampered our understanding of these processes. The biofilm optotracing technology described here is designed to overcome this shortage. A major advantage is that this method enables direct tracing of ECM production *in situ* under a variety of conditions, including liquid cultures and growth on biotic and abiotic surfaces. Acting as structure-responsive chameleons, altered geometry of the highly photostable LCO molecules, due to the structural orientation of the thiophene backbone when bound to a target, is instantly translated into altered intensity and spectral properties of emitted light at given wavelengths. When applied as a non-toxic medium additive, LCOs continuously incorporates into the growing biofilm, emitting target-specific opto-signals immediately as the target is produced. This circumvents the conventional problem of dye penetration into ECM and enables true real-time analysis. By eliminating fixation and washing procedures, the one-step biofilm optotracing technology is ideal for studies of weakly attached and pellicle biofilms. In comparison with the gold standard CV assay that is limited to end-point experiments, our method was shown to be dynamically and molecularly superior, offering simultaneous tracing and differentiation of ECM production in real-time. We demonstrate here the biofilm optotracing technology using two *Salmonella* serovars, the method should be widely applicable to any bacterial species producing amyloid curli protein and cellulose polysaccharide.

Conceptually, the optotracing technology differs not only from traditional dyes, but also from antibody labelling. Whereas specificity of the latter lies in the primary antibody–ligand interaction, the binding pattern of LCO molecules shows a degree of promiscuity. Signal specificity is rather obtained by features such as the exclusive combination of excitation and emission wavelengths for explicit combinations of sensor and target molecules, and the inherent ON/OFF switching that produces markedly increased emission only when target proteins and polysaccharides are detected. The optotracing technology thus represents a one-step process, which eliminates the need of fluorophore-conjugated antibodies or lectins for detection.

We see the reported technology as complementary to current methods in biofilm research. In imaging applications, the optotracing technology helps to overcome the shortage of polysaccharide-targeting antibodies, and supplements the use of lectins. In molecular studies, combining the dynamic optotracing technology with transcriptomic and proteomic studies of end-stage bacterial cultures will promote better understanding of the dynamic regulatory systems governing biofilm formation. One example is curli expression, reported to occur during stationary phase^33–35^ as its biosynthesis depends on the biofilm regulator CsgD, whose expression requires the stationary-phase sigma factor RpoS.^3,36^ Parallel measurements of turbidity and the presence of curli and cellulose during exponential growth in batch culture imply, however, that transcription and translation of ECM factors occur already in mid-exponential phase, since assembled curli fibres and cellulose polysaccharides are detected extracellularly at late exponential/early stationary phase. Dynamic recordings also suggest that compensatory mechanisms modulate biofilm formation, demonstrated by cellulose expression occurring ca 4 h earlier in a curli-negative strain compared to the wt. The molecular mechanisms governing co-regulation of curli and cellulose remains to be elucidated. The biofilm optotracing technology is also well suited for analysing bacterial colonies on solid media. Whereas traditional rdar morphotyping is visually determined on CR- or calcofluor-containing agar plates, LCOs provide an unbiased method for specific determination of ECM components as soon as colonies appear on the LB agar plates. The timesaving aspect is further enhanced, since screening for the biofilm phenotype on LB agar allows colonies originating from a single bacterium to be used immediately for further genetic processing.

As with most technologies in their infancy, the method does have limitations. With the limited set of LCOs and target molecules tested to date, the frequency of target molecules adopting similar geometry, thereby producing overlapping optical spectra, is currently unknown. Also, the presence of unknown molecules in complex media also poses a risk of unexpected binding. This was illustrated when h-HTAA was used to monitor biofilm formation in unwashed cultures, where a myriad of molecules obscured the signals from curli and cellulose. However, this shortcoming was not observed for the chemically related h-FTAA, verifying that a minor chemical modification of the LCO, e.g., varying the spacing between the carboxyl groups along thiophene backbone, can improve the performance of the LCO. Recent studies have also shown that specificity towards distinct biomolecular targets can be achieved by adding proper β-side chain functionalities along the thiophene backbone and that replacement of the central thiophene moiety with other heterocyclic moieties can generate a palette of LCOs with distinct emission profiles covering the visual spectrum.^37,38^ Overall, we foresee that similar chemical modifications of the LCOs will be extremely useful for overpowering potential limitations of the method.

The molecular sensors used in this study belong to the exciting group of electroactive polymers, whose electronic and ionic conductivity only recently has started to be explored in infection research.^39^ The biofilm optotracing technology presented here represents the first photophysical application of conducting polymers in infection research, thereby widening the opportunities of organic conducting polymers in infection biology. Ongoing work in our group is aimed at defining further LCOs binding characteristics to different elements of the ECM as well as across a wide-range of bacterial species.

## Methods

### h-HTAA and h-FTAA

The luminescent conjugated oligothiophene h-HTAA and h-FTAA were synthesised as previously described^16,17,40^ according to the scheme in Supplementary Figure 7. Stock solutions (1.5 mmol/l) prepared in ^D^H_2_O were maintained in the fridge. Both LCOs were used at 3 μmol/l final concentration, which is predicted to be in excess based on the original article.^16^

### Bacterial strains, media, generation time and viability assay

Bacterial strains (Supplementary Table 1) were routinely cultured on Luria-Bertani (LB) agar or in LB broth at 37 °C. LB without (w/o) salt was used to promote biofilm formation at 28 °C. The GFP-encoding plasmid p2777, purified from *S.* Typhimurium NCTC 12023 by QIAprep Spin kit (Qiagen, Hilden, Germany), was passaged via transfer strain ARD83 into *S.* Enteritidis 3934 wt and isogenic strains by electroporation.^41^ Positive clones were selected on LB plates with ampicillin (100 μg/ml). Generation time was assessed by diluting cultures of *S.* Enteritidis 3934 at OD_600_=0.6, to 10^5^ CFU/ml in LB. Following transfer of 70 ml to separate flasks, 210 μl of the stock solutions of h-FTAA, and h-HTAA or of PBS was added. OD_600_ of cultures incubated at 37 °C, 230 r.p.m., was recorded every 10 min to define generation time. Viable count was obtained by spread plating 100 μl withdrawn from the culture at indicated times onto LB agar plates.

### Congo red assay

Morphotypes based on curli and cellulose expression were observed after growth at 28 °C for 1–3 days on LB plates w/o salt, supplemented with Congo red (40 μg/ml, Sigma-Aldrich, Stockholm, Sweden) and Coomassie brilliant blue G-250 (20 μg/ml, Sigma) dissolved in 70% ethanol (Kemetyl AB, Haninge, Sweden).

### Inclined coverslip assay

Exponentially growing cultures (37 °C, 230 r.p.m.) of indicated strains were harvested at OD_600_=0.6, diluted to 10^5^ CFU/ml in LB w/o salt, dispensed (8 ml aliquots) in six-well plates (Sarstedt, Numbrecht, Germany) with inclined glass coverslips (24×24 mm, VWR International, Stockholm, Sweden), and incubated for 48 h at 28 °C to allow biofilm formation. Removed coverslips were washed twice in PBS before and after 1 h fixation in 4% formaldehyde, then immersed in 3 μmol/l h-HTAA or h-FTAA in PBS. Following 30 min incubation in the dark, coverslips were washed twice in PBS, then prepared for microscopy analysis. In negative controls, coverslips were immersed in PBS without added LCOs. Biofilm formed on the surface at the air–liquid interface was analysed on coverslips immobilised (Vectashield, VWR International) onto glass slides. The 20× dry objective on an Olympus FV1000 confocal microscope (Olympus, Stockholm, Sweden) was used with preset detection settings for transmission, GFP (h-HTAA) and Cy3 (h-FTAA) fluorescence.

### Small-volume 96-well biofilm assays

Semi-high-throughput biofilm recordings were achieved by downsizing the conventional 24-well plate format to the scale of a 96-well plate. Bacterial overnight cultures (37 °C, 230 r.p.m.) were diluted in LB w/o salt to 10^5^ CFU/ml. Aliquots were made to which h-HTAA or h-FTAA (3 μmol/l final concentrations), or PBS was added. Similar mixtures without bacteria were used as negative controls. 50 μl of each mixture, dispensed in triplicates into tissue culture treated 96-well plates (Sarstedt, Hilden, Germany) were incubated at 28 °C. To prevent desiccation, sterile water (300 μl) was added to each unused well and to fill the entire inter-well region (Supplementary Figure 2). At selected time points, supernatants were discarded and wells were rinsed twice with 150 μl PBS prior to analysis.

Conventional biofilm analysis was performed by adding 100 μl, 0.4 % Crystal violet (CV) (Sigma-Aldrich) to wells with PBS originally added to the culture. After 10 min in room temperature, wells were rinsed twice with PBS. Surface-attached CV was released following incubation of wells in 125 μl 99.5% EtOH for 15 min at room temperature. 100 μl from each well was transferred to a new plate and the CV absorbance was recorded at 540 nm in a Synergy Mx Monochromator-Based Multi-Mode Microplate Reader (Biotek, Bad Friedrichshall, Germany).

In parallel, biofilm was quantified in wells with cultures originally supplemented with h-HTAA and h-FTAA. Direct comparison of biofilm amounts in this and the CV assay was achieved by exposing LCO-supplemented wells to exactly the same experimental procedures, using PBS instead of CV and EtOH. In the final steps, 100 μl from each well was transferred into a new plate, and optical recording of LCOs was achieved by excitation at 405 nm and emission detected at 545 nm (h-HTAA) and 556 nm (h-FTAA) using the plate reader.

### LCO-based biofilm analysis under non-disruptive conditions

Bacterial cultures supplemented with h-HTAA and h-FTAA (3 μmol/l final concentration) were seeded into the small-volume 96-well biofilm assay as described. Non-disruptive biofilm recording was achieved by positioning the seeded plate in a Synergy Mx Monochromator-Based Multi-Mode Microplate Reader (Biotek), and recording excitation spectra of the growing culture at indicated times. Excitation spectra were collected at 300–500 nm, with emission detected at 545 nm. Curli-specific emission spectra were collected by exciting cultures at 405 nm and reading emission at 500–700 nm. Cellulose-specific emission spectra were collected by excitation at 500 nm and emission recorded at 520–700 nm.

### LCO-based cellulose detection

Serial two-fold dilutions, ranging from 5–0.04 mg/ml, of microcrystalline cellulose (Sigma-Aldrich) were prepared in sterile ^D^H_2_O. After mixing equal volumes of cellulose suspensions and h-FTAA (3 μmol/l final concentration), 50 μl of each mix was transferred to a 96-well plate, which was placed in a Synergy Mx Monochromator-Based Multi-Mode Microplate Reader (Biotek). Excitation spectra in the range 300–500 nm were collected with emission detected at 545 nm.

### Morphotyping of biofilms

The conventional method for Congo red-based rdar morphotyping^42,43^ was adapted to enable LCO-based spectral morphotyping of biofilms. Cultures (LB medium, 37 °C, 230 r.p.m.) of the 3934 wt strain, and isogenic mutant strains Δ*bcsA*, Δ*csgA*, and Δ*csgD* harvested in exponential phase (OD_600_=0.6) were diluted by a factor of 10^7^ in PBS. A volume of 25 μl of each dilution was spotted on Congo red assay plates^42^ and LB agar plates w/o salt, and incubated at 28 °C for 48 h. The rdar morphology was documented on the Congo red plates. For LCO-based spectral morphotyping, single colonies from the LB plates were harvested with a loop and dissolved in 500 μl PBS. After four cycles of 5 s sonication and 10 s pause, 25 μl from each suspension was mixed with 25 μl of 6 μmol/l h-FTAA and transferred to a 96-well plate. As control, h-FTAA added to PBS or to a cellulose (Sigma-Aldrich) suspension (0.1 mg/ml in PBS) was used. Excitation spectra in the range 300–500 nm were collected with emission detected at 545 nm.

To monitor the kinetics of ECM production, 100 μl from an exponential (OD_600_=0.6) culture of 3934 wt was diluted by a factor of 10^5^, then spread plated on Congo red assay plates^42^ and LB agar plates w/o salt, and incubated at 28 °C. At days 1, 2 and 3, the rdar morphology of single-cell colonies on Congo red plates was documented. In parallel, single-cell colonies on LB plates were harvested with a loop and dissolved in 500 μl PBS. After four cycles of 5 s sonication and 10 s pause, 25 μl from each suspension was mixed with 25 μl of 6 μmol/l h-FTAA and transferred to a 96-well plate. As control, h-FTAA added to PBS or to a cellulose (Sigma-Aldrich) suspension (0.1 mg/ml in PBS) was used. Excitation spectra in the range 300–500 nm were collected with emission detected at 545 nm.

### Real-time kinetics of ECM expression during bacterial growth in liquid cultures

Cultures (10^5^ CFU/ml) of strain 3934 wt, and isogenic mutants Δ*bcsA*-, Δ*csgA*- and Δ*csgD-*containing plasmid p2777 were prepared in LB w/o salt from an overnight culture.^44^ After addition of h-FTAA (3 μmol/l final concentration), 50 μl aliquots were seeded into four parallel 96-well plates, which were placed in a 28 °C incubator. Every hour, one plate was positioned in a Synergy Mx Monochromator-Based Multi-Mode Microplate Reader (Biotek), allowing 4 h intervals between scans of individual plates to minimise culturing disturbances. Bacterial culture density was monitored by GFP fluorescence intensity (*λ*_Ex_ 445 nm, *λ*_Em_ 510 nm) for a period of 48 h. Simultaneously, curli (*λ*_Ex_ 405 nm, *λ*_Em_ 556 nm) and cellulose (*λ*_Ex_ 500 nm, *λ*_Em_ 600 nm) were recorded. A time series was obtained by combining readings from the four plates. Relative fluorescence units of GFP and of h-FTAA binding to curli and cellulose are presented in the same graphs to visualise the hourly change in signals.

### Live fluorescence confocal imaging of h-FTAA-stained biofilm ECM

h-FTAA (3 μmol/l final concentration) was added to cultures (10^5^ CFU/ml) of strain 3934 wt p2777 and *S.* Typhimurium strain 14028 *ssaG::gfp^+^* prepared in LB w/o salt. A volume of 8 ml aliquots were transferred into six-well plates with inclined glass coverslips to allow biofilm formation at the air–liquid interface during 48 h incubation in 28 °C. Biofilm formed on the removed coverslips, washed twice with PBS, were analysed by fluorescence confocal microscopy (FV1000 confocal microscope, Olympus) using a UPLSAPO 40× 2 (NA 0.95) lens and a UPLSAPO 60× W (NA 1.2) water immersion lens (Olympus). In parallel, control experiments were performed using bacteria cultured in the absence of h-FTAA, or h-FTAA added to strain 3934 wt lacking the GFP expressing plasmid.

### Intracellular cellulose expression stained by h-FTAA

Macrophage-like cell line RAW264.7 (TIB-71; ATCC, Manassas, VA, USA) was propagated in supplemented RPMI 1640 (Gibco, Paisley, UK)^45^ and the epithelial cell line CRL-4031 (ATCC) in supplemented DMEM/F12 (Gibco, Paisley, UK)^46^ as described. *S.* Typhimurium strain 14028 *ssaG::gfp^+^* grown in LB, 37 °C, was harvested at OD_600_=0.6, resuspended in respective cell culture media, and used at multiplicity of infection (MOI)=10 to infect RAW264.7 and CRL-4031 cells seeded on round coverslips in 24-well plates. Media were exchanged after 90 min to gentamicin-containing (50 μg/ml, Sigma-Aldrich) media, and incubation proceeded for 30 min to kill extracellular bacteria. After a shift to maintenance medium (10 μg/ml gentamicin) with infection proceeding for 90 min, supernatants were discarded. Cells were rinsed twice in PBS, fixed in 4% paraformaldehyde for 1 h, and rinsed twice in PBS. Fixed cells were then treated with ice-cold acetone for 4 min, rinsed twice in PBS and stained with Hoechst 33342 (Life Technologies, Stockholm, Sweden), Alexa Fluor 647 Phalloidin (Life Technologies) and h-FTAA (3 μmol/l) in PBS for 1 h. Coverslips washed twice in PBS were mounted on microscope slides using Prolong Gold (Life Technologies). Fluorescence confocal microscopy (FV1000 confocal microscope (Olympus), using a UPLSAPO 60× W (NA 1.2) water immersion lens (Olympus) was performed using optical settings for Hoechst 33342, GFP^+^ (*λ*_Ex_ 473 nm, *λ*_Em_ 490–540 nm) and h-FTAA (*λ*_Ex_ 473 nm, *λ*_Em_ 590–655 nm). The imaging processing software Fiji (ImageJ, Bethesda, MD, USA) was used for image analysis.^47^ Three-dimensional projections were generated by brightest point projection of image stacks (10 μm stack of 0.5 μm steps) and processed into.MOV files of 15 fps.

Mice (6–8 weeks BALB/c, Taconic Europe, Lille Skensved, Denmark) were intraperitoneally injected with 10^4^ CFU *S.* Typhimurium strain 14028 *ssaG::gfp^+^*^48^ in 100 μl PBS, and the control group with PBS only. Livers excised from mice 3 days post infection were cut in 5 mm pieces that were immediately snap-frozen in a slurry of dry ice/70% ethanol. Ten micrometre sections were treated with ice-cold acetone for 4 min and rinsed twice in PBS. Sections were stained with Hoechst 33342, h-FTAA (3 μmol/l) in PBS and/or Alexa Fluor 647 Phalloidin (Life Technologies) for 1 h, washed twice with PBS, and mounted in Prolong Gold (Life Technologies). Fluorescence confocal microscopy (FV1000 confocal microscope, Olympus) was performed using the same optical settings as for infected cell lines. Mice were housed at MTC animal facility (Karolinska Institutet, Stockholm, Sweden) in accordance with institutional and national guidelines (ethical permit N491/11, Stockholm Norra Djurförsöksetiska Nämnd).

### Statistical analysis

All data were organised and processed with GraphPad Prism 6 (Graphpad Software, La Jolla, CA, USA). To determine the significance of the observed difference in cellulose expression kinetics, cellulose-specific relative fluorescence units collected from growing biofilm cultures of strain 3934 wt+P2777 and *ΔcsgA*+P2777 were first normalised to eliminate differences in intensities such that the strongest and weakest relative fluorescence units was represented by 100% and 0%, respectively. Significance analysis was then performed by two-way Anunivariate analysis of variance (ANOVA) with multiple comparisons, in which the mean of cells from each row from each strain is compared.
