# Supplementary material for: Real-time optotracing of curli and cellulose in live Salmonella biofilms using luminescent oligothiophenes
Source: NPJ Biofilms Microbiomes. 2016 Nov 23;2:16024–. doi: 10.1038/npjbiofilms.2016.24 (PMC5515270; doi:10.1038/npjbiofilms.2016.24)
Supplement: Supplementary Information [file npjbiofilms201624-s1.pdf]

SUPPLEMENTARY INFORMATION

Real-time opto-tracing of curli and cellulose in live *Salmonella* biofilms using luminescent oligothiophenes

Ferdinand X. Choong[1], Marcus Bäck[2], Sara Fahlén[1], Leif B. G. Johansson[2], Keira Melican[1], Mikael Rhen[1,3], K. Peter R. Nilsson[2], and Agneta Richter-Dahlfors[1]*

[1] Swedish Medical Nanoscience Center, Department of Neuroscience, Karolinska Institutet, SE-17177, Stockholm, Sweden

[2] Division of Chemistry, Department of Physics, Chemistry and Biology, Linköping University, SE-581 83 Linköping, Sweden

[3] Department of Microbiology, Tumor and Cell biology, Karolinska Institutet, SE-17177 Stockholm, Sweden

Supplementary Figure 1

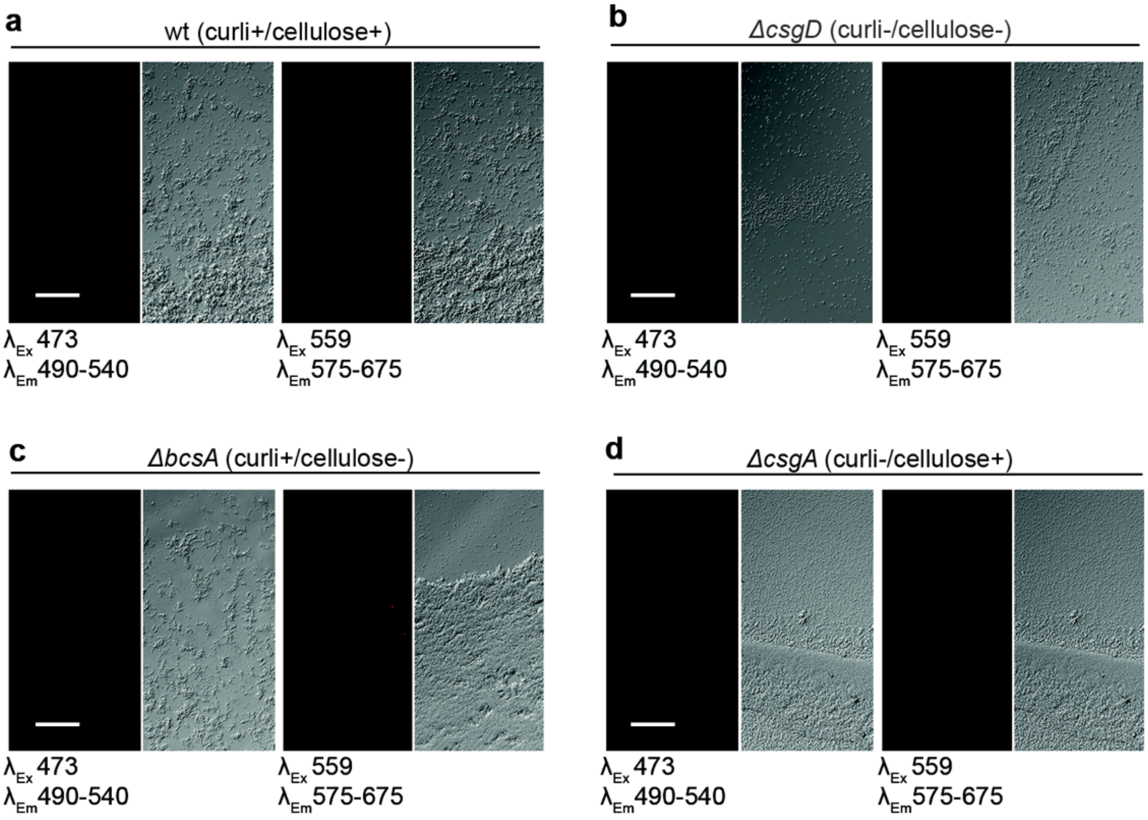

Unstained biofilms show no autofluorescence.

Fluorescence confocal (left) and transmission (right) microscopy of unstained biofilms from strains 3934 (**a**) wt, (**b**) Δ*csgD*, (**c**) Δ*bcsA,* and (**d**) Δ*csgA*. Fluorescence is recorded at λ_{Ex} 473 nm, λ_{Em} 490 – 540 nm, and λ_{Ex} 559 nm, λ_{Em} 575 - 675 nm. These settings represent the same optical settings as used in Fig. 1. Single optical sections are shown. Scale bar = 50 µm.

Supplementary Figure 2

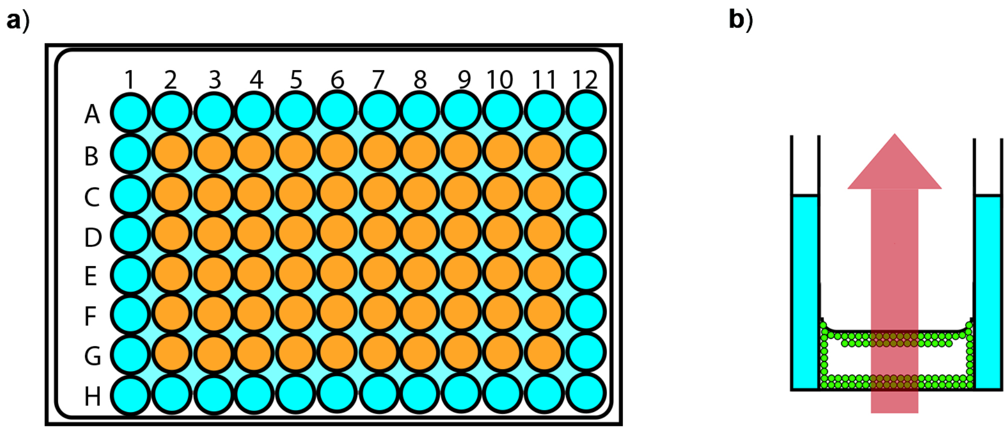

Small-volume 96-well assay for fluorometric recording of biofilm formation.

(**a**) Overview of the 96-well plate layout with bacterial cultures in indicated wells (yellow). To maintain humidity and prevent desiccation in the small-volume (50 µl) bacterial cultures during incubation, the outermost wells and in the inter-well regions were filled with sterile water (blue). (**b**) Schematic presentation of an individual well. While biofilm-forming bacteria form biofilm (green) on all interfaces of the well, only the biofilm formed at the air/liquid (pellicle) and liquid/solid interface in the path of the detector (red arrow) are recorded.

Supplementary Figure 3

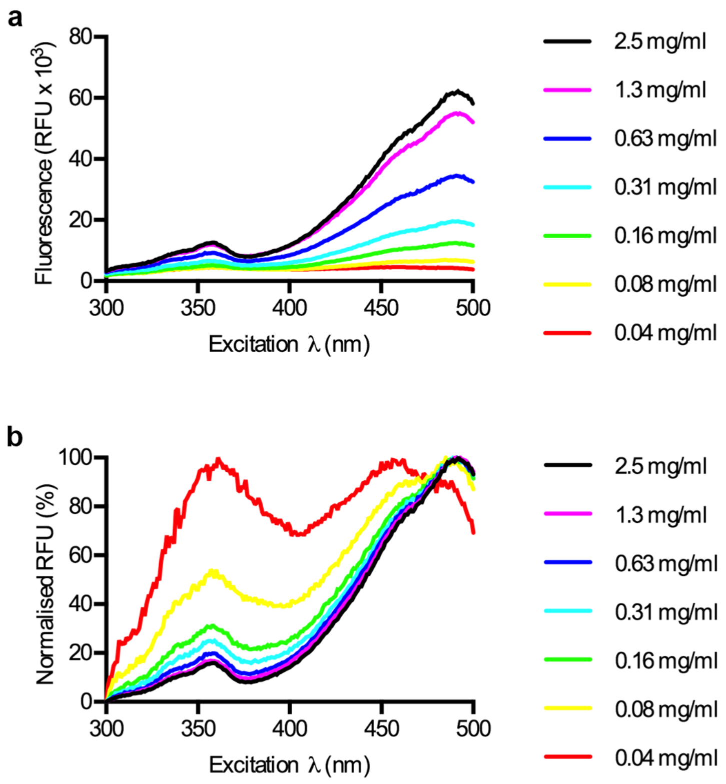

Binding of h-FTAA to cellulose generates a distinct, highly sensitive, spectral signature.

(**a**) Excitation spectra of h-FTAA binding to cellulose at indicated amounts of polysaccharide. (**b**) Normalized fluorescence spectra show a distinct cellulose peak at 490 nm in samples containing ≥ 0.08 mg/ml cellulose. Data represents n:1 of 3. RFU = relative fluorescence units.

Supplementary Figure 4

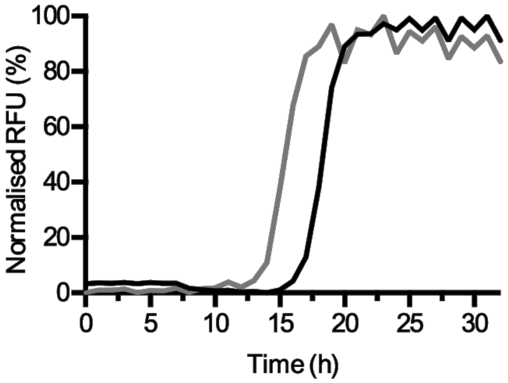

The kinetics of cellulose production is influenced by the curli producing ability of biofilm-forming strains.

Normalized spectra of h-FTAA-supplemented small-volume 96-well assays of the 3934 wt (black) and Δ*csgA* mutant (grey) strains reveals that the kinetics of cellulose formation is influenced by the strains' ability to produce curli. Extracellular cellulose appears 4 h earlier in the curli-deficient mutant compared to the curli-producing wt strain. Statistical analysis of the data is presented in Supplementary Table 2. Data represents n:1 of 3. RFU = relative fluorescence units.

Supplementary Figure 5

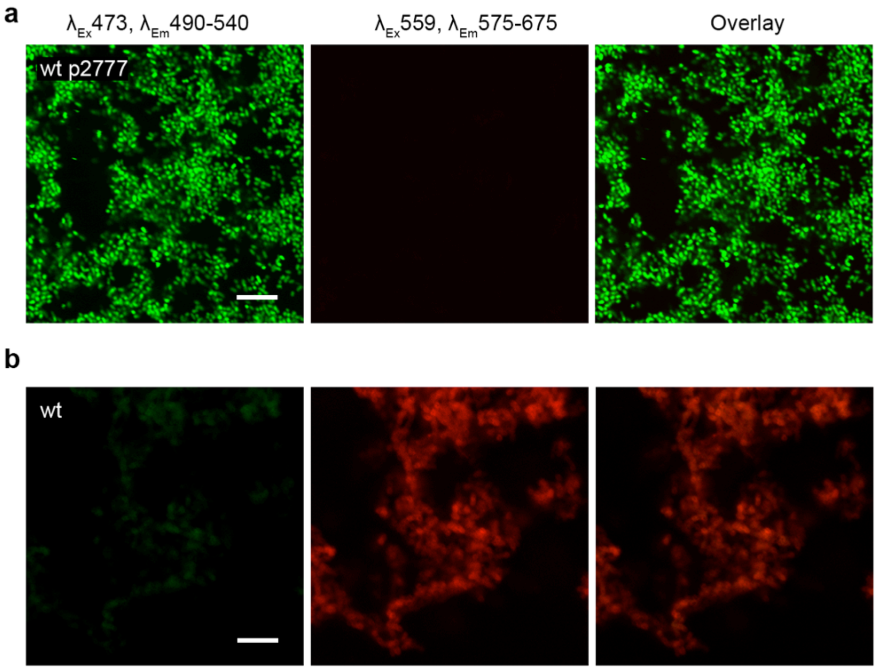

Analysis of fluorescence pattern of *S.* Enteritidis biofilms.

Fluorescence confocal microscopy of unfixed biofilms formed by (**a)** the GFP-expressing strain 3934 wt p2777 in the inclined coverslip setup clearly shows bacterial cells in the green channel (λ_{Ex} 473 nm, λ_{Em} 490 – 540 nm). No fluorescence is observed in the red channel (λ_{Ex} 559 nm, λ_{Em} 575 – 675 nm). (**b**) Biofilm from the wt strain lacking GFP expression grown in the presence of h-FTAA shows expected ECM staining in the red channel, as well as a degree of bleed-through into the green channel. This weak signal is due to the inherent wide and strong emission from h-FTAA. Single optical sections are shown. Scale bar = 10 μm.

Supplementary Figure 6

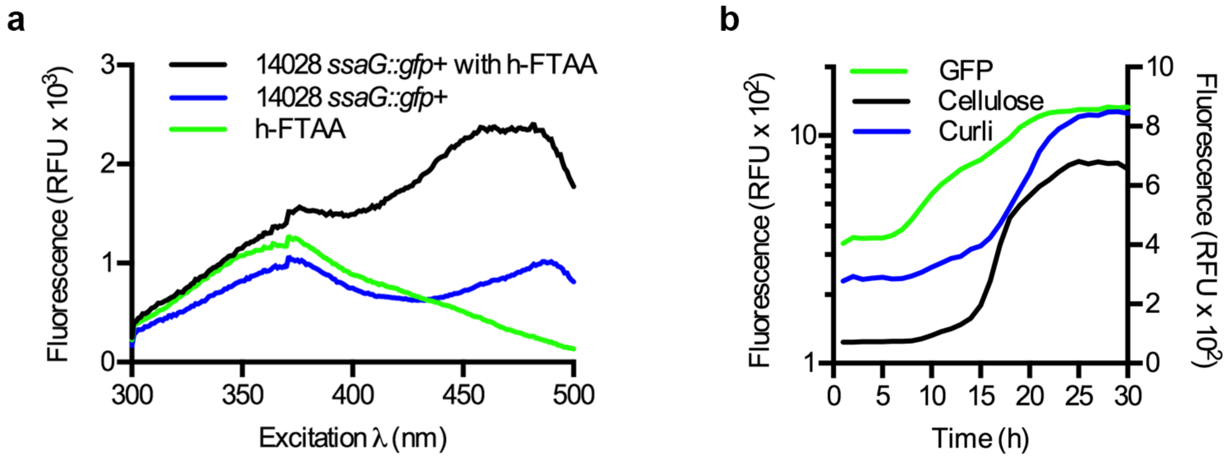

h-FTAA identifies cellulose produced by *S.* Typhimurium.

(**a**) Spectral profiling of biofilm formed by *S*. Typhimurium, strain 14028 *ssaG::gfp*+ (black) grown for 48 h in h-FTAA supplemented small-volume 96-well assay shows the presence of curli (increased fluorescence at ~ 405 nm) and cellulose (increased fluorescence at ~ 480 nm). In the absence of h-FTAA (blue), bacterial GFP expression is observed as an excitation peak at 490 nm. No fluorescence increase in the 405 nm and 480 nm range is observed in the control containing only h-FTAA (green). (**b**) Real-time recording of GFP (left y-axis) and h-FTAA (right y-axis) fluorescence during growth of *S*. Typhimurium, strain 14028 *ssaG::gfp*+ in the small-volume 96-well assays. The steep increase in cellulose (black) and curli (blue) signals is observed during late exponential / early stationary phase of bacterial growth. Data represents n:1 of 3. RFU = relative fluorescence units.

.

Supplementary Figure 7

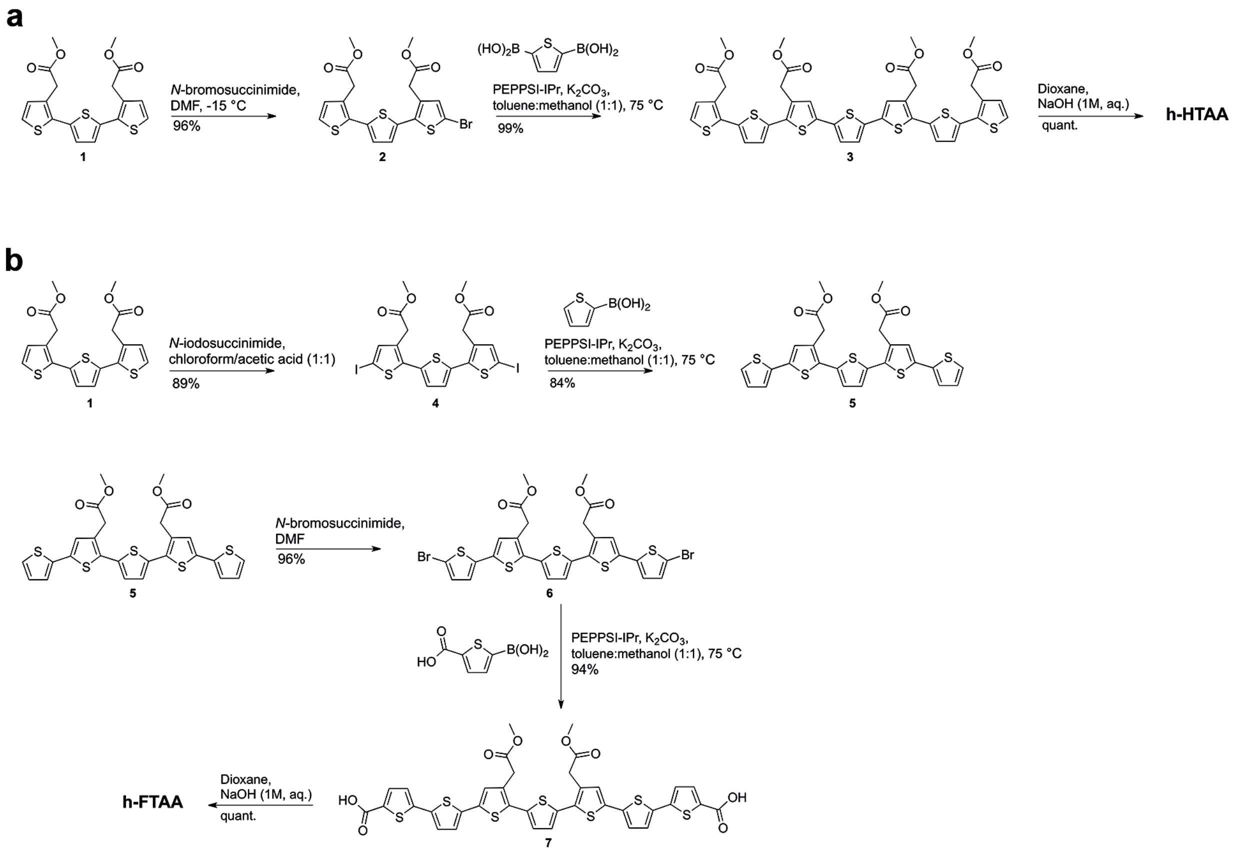

Schematic of the chemical synthesis of LCOs used in this study.

(**a**) The sequential synthesis of h-HTAA is schematically depicted based on details presented in reference 16 and 40. (**b**) The h-FTAA synthesis scheme is described in detail in references 16 and 17.

Supplementary Table 1.

Strains and plasmids used in this study

Strains or Plasmids		Characteristics	Source
Strains	Alternate name		
S. Enteritidis			
ARD79	3934	Wild-type clinical isolate	*Reference 10*
ARD80	3934 *ΔcsgD*	3934 *ΔcsgD*::KmR	*Reference 23*
ARD81	3934 *ΔcsgA*	3934 *ΔcsgA*::KmR	*Reference 24*
ARD82	3934 *ΔbcsA*	3934 *ΔbcsA*::KmR	*Reference 10*
ARD204	3934 p2777	3934 expressing green fluorescence protein, AmpR, KmR	This study
ARD205	3934 *ΔbcsA* p2777	3934 *ΔbcsA* expressing green fluorescence protein, AmpR, KmR	This study
ARD206	3934 *ΔcsgA* p2777	3934 *ΔcsgA* expressing green fluorescence protein, AmpR, KmR	This study
ARD207	3934 *ΔcsgD* p2777	3934 *ΔcsgD* expressing green fluorescence protein, AmpR, KmR	This study
ARD83	LB5010	*metA22, metE551, trpD2, ilv452, hsdLT6, hsdSA29, hsdSB, galE*	*Reference 44*
S. Typhimurium			
	14028 *ssaG::gfp+*	14028 expressing green fluorescent protein	*Reference 50*
Plasmids			
p2777		Vector for green fluorescent protein expression and ampicillin resistance	*Reference 47*

Supplementary Table 2.

Statistical analysis of the kinetics of cellulose production during bacterial growth.

Time (h)	Mean difference (RFU)	95% confidence interval of difference	Significance
0	3.291	-3.318 to 9.900	No
1	2.687	-3.922 to 9.296	No
2	2.643	-3.965 to 9.252	No
3	2.328	-4.280 to 8.937	No
4	3.233	-3.375 to 9.842	No
5	2.894	-3.715 to 9.502	No
6	2.839	-3.769 to 9.448	No
7	1.821	-4.788 to 8.429	No
8	1.454	-5.155 to 8.063	No
9	-0.3926	-7.001 to 6.216	No
10	-1.072	-7.681 to 5.537	No
11	-3	-9.609 to 3.609	No
12	-1.652	-8.260 to 4.957	No
13	-4.044	-10.65 to 2.565	No
14	-10.92	-17.53 to -4.314	Yes (****)
15	-36.2	-42.81 to -29.59	Yes (****)
16	-63.37	-69.98 to -56.76	Yes (****)
17	-72.66	-79.27 to -66.05	Yes (****)
18	-50.77	-57.38 to -44.16	Yes (****)
19	-22.71	-29.32 to -16.10	Yes (****)
20	5.853	-0.7554 to 12.46	No
21	-1.598	-8.206 to 5.011	No
22	0.1942	-6.414 to 6.803	No
23	-2.797	-9.405 to 3.812	No
24	8.446	1.837 to 15.05	Yes (**)
25	4.655	-1.953 to 11.26	No
26	3.743	-2.866 to 10.35	No
27	3.264	-3.345 to 9.872	No
28	6.424	-0.1847 to 13.03	No
29	6.781	0.1720 to 13.39	Yes (*)
30	6.599	-0.009455 to 13.21	No
31	7.117	0.5088 to 13.73	Yes (*)
32	7.637	1.029 to 14.25	Yes (**)

The table shows a statistical analysis of data presented in Supplementary Figure 4. The mean difference in fluorescence between the wt and Δ*csgA* mutant strain analyzed in triplicate at each time point is shown, along with the statistical significance. Data represents n:1 of 3. RFU = relative fluorescence units. P-values (*) = $P \leq 0.05$, (**) = $P \leq 0.01$, (****) = $P \leq 0.0001$.

Supplementary Movies 1 and 2

Visualization of intracellular *S.* Typhimurium in cell cultures.

3D projections of fluorescence confocal microscopy images sets of CRL-4031 epithelial cells (**Supplementary Movie 1**) and RAW264.7 macrophages (**Supplementary Movie 2**) infected with *S.* Typhimurium strain 14028 *ssaG:gfp+* (green). Image sets contain 23 slices with 0.49 µm steps and 21 slices with 0.5 µm steps respectively, and are combined by brightest point projection. Staining with Hoechst 33324 and Alexa Fluor® 647 Phalloidin shows the nuclei (blue) and the actin cytoskeleton (grey) of each cell type. Scale bar = 10 µm.
